# Supplementary material for: An Integrated Linkage Map of Three Recombinant Inbred Populations of Pea (Pisum sativum L.)
Source: Genes (Basel). 2022 Jan 22;13(2):196. doi: 10.3390/genes13020196 (PMC8871737; doi:10.3390/genes13020196)
Supplement: Supplementary file 1 [file genes-13-00196-s001.zip › Figures S1-S6.pdf]

## Supplementary Figures

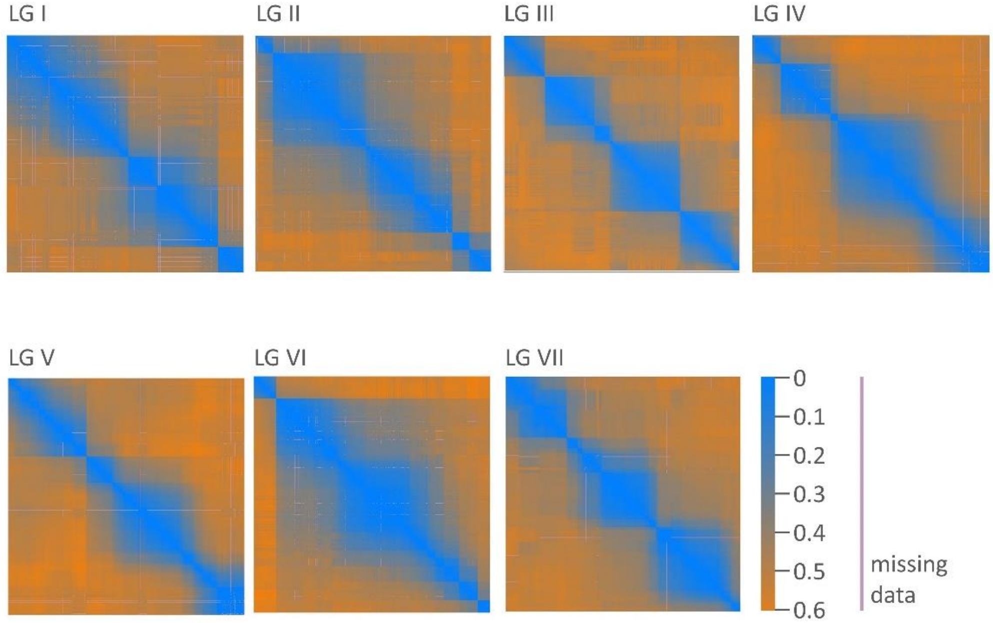


**Figure S1**. Heat maps of pairwise inter-marker distances per linkage group. Each panel corresponds to one linkage group and within each panel each row and column corresponds to a single marker. The markers are ordered left to right and top to bottom according to their position in the linkage map. The diagonal line therefore corresponds to markers that are identical and necessarily have an inter marker distance of zero. Adjacent to the LG VII panel the scale for the heatmap colors is shown. Discontinuities in color correspond to unusually large intervals. The color corresponding to pairwise comparisons which cannot be made i.e. where at least one of the marker pairs is scored in only one population and the other marker is not scored in that population is indicated 'missing data', see Table 2 .

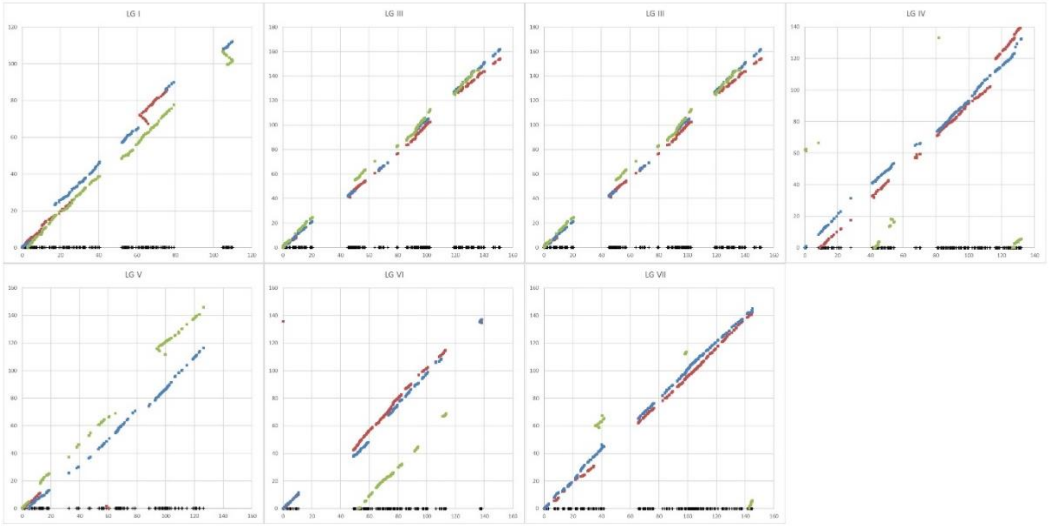

**Figure S2**. Comparing individual genetic maps with the integrated map. For each linkage group marker position in the integrated map (in $cM_{Haldane}$) is plotted as '+' on the x axis while the corresponding map position (calculated using ASMap [5]) in the three individual maps is plotted on the y axis (BK in red, EK in blue and BE in green).

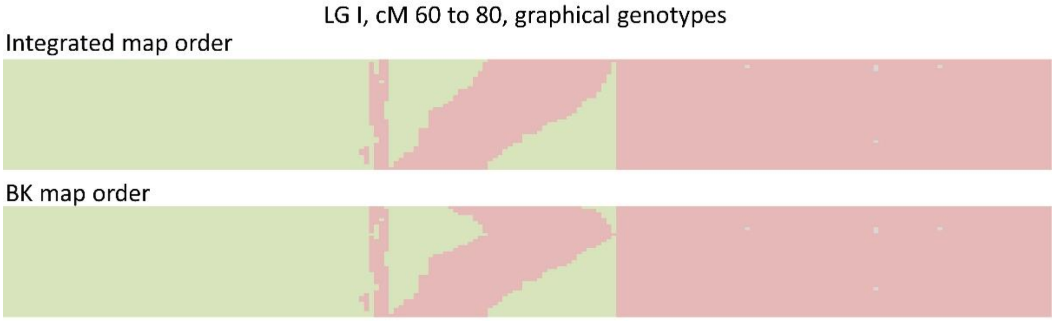


**Figure S3**. An inverted map segment in the BK linkage group I map. Graphical genotypes are shown for the region of LG I where the order of markers in the BK population proposed by ASMap differs from the marker order in the BEK integrated map and the ASMap derived order for the BE population. Markers are ordered top to bottom and RILs ordered left to right to group lines according to genotype. The upper panel has the markers in the BEK map order while the lower panel shows the same markers in the ASMap order showing the double recombinants proposed by ASMap.

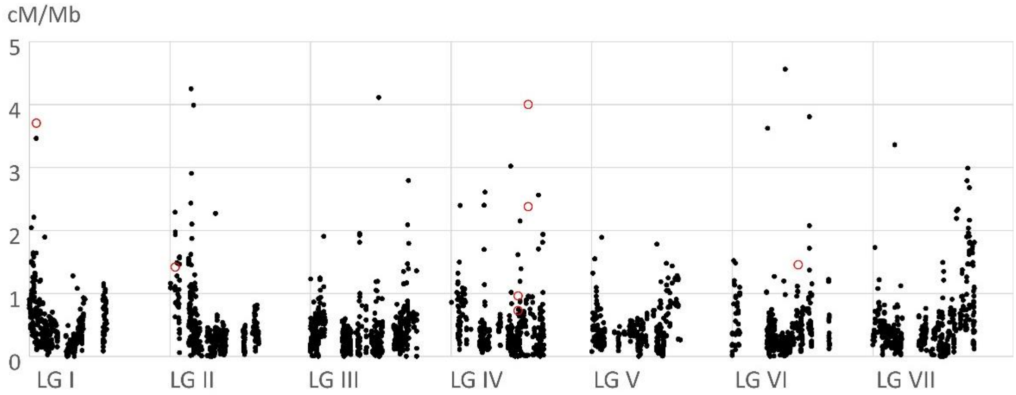


**Figure S4**. Recombination rates. For each linkage group recombination rates were calculated as described in Table 4. These values are plotted (black dots) on the y axis and the x-axis value corresponds to the marker in the middle of the pair being compared. Seven values are large and these are marked with a red open circle where the recombination rate is tenfold higher than shown by the y-axis scale.

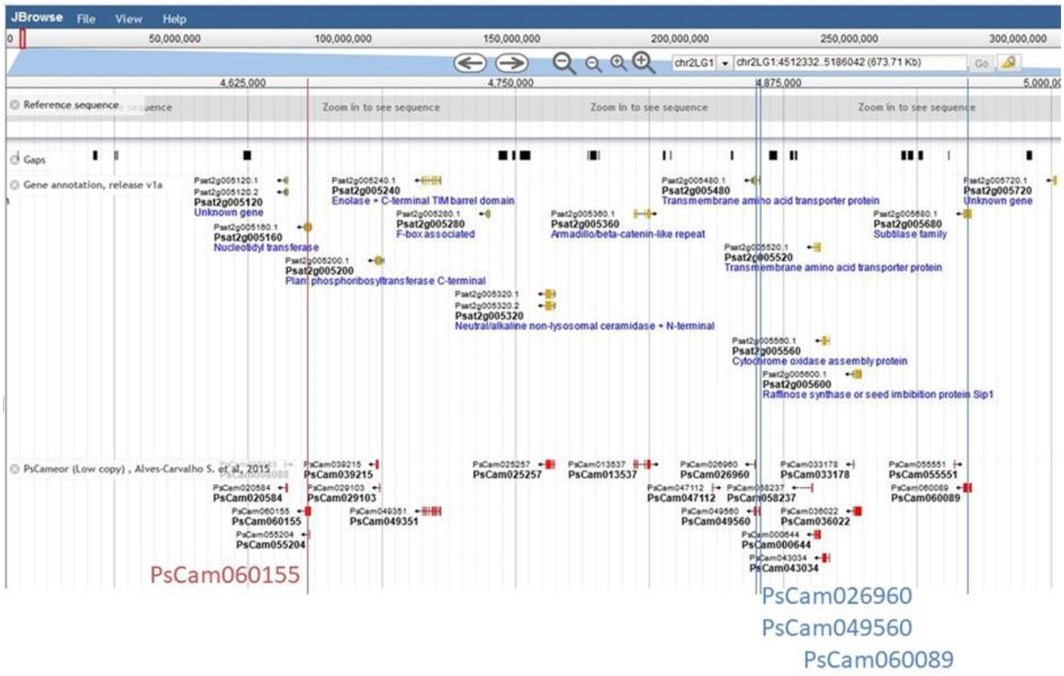

**Figure S5**. Candidate genes for a major seed weight QTL. Annotated screen shot of the region of the QTL peak for mean seed weight on linkage group I, detected in the BK (red) and EK (blue) sub-populations. The markers in PsCam026960, 049560 and 060089 all co-segregate (and with PsCam014211, PsCam042135 which segregate in the BE sub-population and are just to the right of this screen shot). There are five RILs which have a recombination event between PsCam060155 and PsCam026960, two in the EK sub-population and three in the BK sub-population.

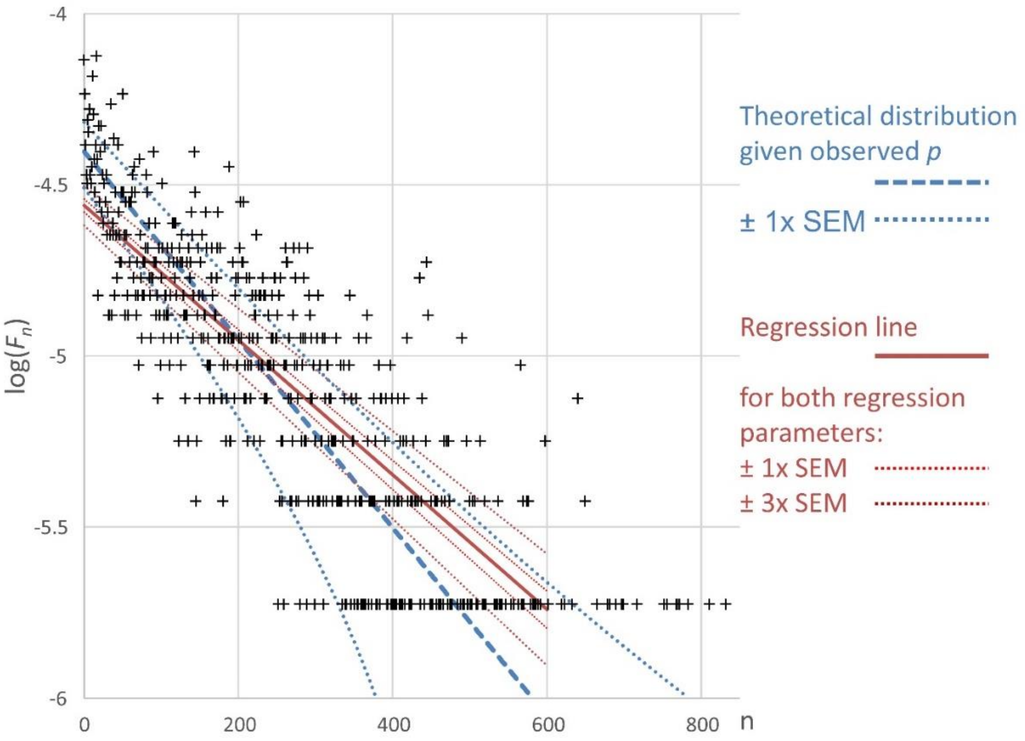


**Figure S6.** Frequency distribution of the number of intervals between recombination events. See text for explanation of the graph. Number of successive intervals between recom-bination events (*n*); log($F_n$) common logarithm of the frequency of intervals of length *n*.
